# Inhibition of Histone H3K9 Methylation by BIX-01294 Promotes Stress-Induced Microspore Totipotency and Enhances Embryogenesis Initiation

**DOI:** 10.3389/fpls.2017.01161

**Published:** 2017-06-29

**Authors:** Eduardo Berenguer, Ivett Bárány, María-Teresa Solís, Yolanda Pérez-Pérez, María C. Risueño, Pilar S. Testillano

**Affiliations:** Pollen Biotechnology of Crop Plants Laboratory, Biological Research Center, Centro de Investigaciones Biológicas – Consejo Superior de Investigaciones Científicas (CIB-CSIC)Madrid, Spain

**Keywords:** stress-induced microspore reprogramming, cell totipotency, microspore embryogenesis, histone methylation, H3K9me2, BIX-01294, rapeseed, barley

## Abstract

Microspore embryogenesis is a process of cell reprogramming, totipotency acquisition and embryogenesis initiation, induced *in vitro* by stress treatments and widely used in plant breeding for rapid production of doubled-haploids, but its regulating mechanisms are still largely unknown. Increasing evidence has revealed epigenetic reprogramming during microspore embryogenesis, through DNA methylation, but less is known about the involvement of histone modifications. In this study, we have analyzed the dynamics and possible role of histone H3K9 methylation, a major repressive modification, as well as the effects on microspore embryogenesis initiation of BIX-01294, an inhibitor of histone methylation, tested for the first time in plants, in *Brassica napus* and *Hordeum vulgare*. Results revealed that microspore reprogramming and initiation of embryogenesis involved a low level of H3K9 methylation. With the progression of embryogenesis, methylation of H3K9 increased, correlating with gene expression profiles of *BnHKMT SUVR4-like* and *BnLSD1-like* (writer and eraser enzymes of H3K9me2). At early stages, BIX-01294 promoted cell reprogramming, totipotency and embryogenesis induction, while diminishing bulk H3K9 methylation. DNA methylation was also reduced by short-term BIX-01294 treatment. By contrast, long BIX-01294 treatments hindered embryogenesis progression, indicating that H3K9 methylation is required for embryo differentiation. These findings open up new possibilities to enhance microspore embryogenesis efficiency in recalcitrant species through pharmacological modulation of histone methylation by using BIX-01294.

## Introduction

Plant cells are characterized by their high plasticity and capacity to become totipotent and pluripotent cells that can initiate embryogenesis and organogenesis in response to several stimuli. After external stress signals *in vitro*, the microspore can reprogram to become a totipotent cell that develops into an embryo and subsequently an entire plant. Therefore, the totipotent microspore acquires embryogenic competence to give rise to a whole plant, which is formed by multiple cell types, tissues and organs (Bárány et al., 2005; Verdeil et al., 2007; Grafi et al., 2011b; Prem et al., 2012). The resulting haploid and double-haploid plants are important biotechnological tools in plant breeding as they represent a source of new genetic variability, fixed in complete homozygous plants and obtained in only one generation step (reviewed in Maluszynski et al., 2003). The mechanisms underlying the induction of de-differentiation of a somatic cell and its conversion into a totipotent embryogenic cell are still elusive. Numerous genes of the new developmental program have to be repressed and/or activated during plant cell reprogramming and totipotency acquisition (Finnegan et al., 2000). Increasing evidence indicates that changes in global genome organization and remodeling of chromatin characterize the initiation and progression of development and differentiation processes in both plants and animals (Kouzarides, 2007); several reports have related totipotency of cells to an open chromatin conformation characterized by large nuclei and homogenous euchromatin (Grafi et al., 2011b). Nevertheless, the molecular determinants that regulate reprogramming and totipotency acquisition/establishment, leading to embryogenesis of plant somatic cells, remain poorly understood.

Previous studies have shown modifications in global DNA methylation that accompanied the change in developmental program of the microspore toward embryogenesis, in very different plant species, like *Brassica napus*, *Hordeum vulgare*, *Quercus suber* and *Q. alba* (Solis et al., 2012; Testillano et al., 2013; El-Tantawy et al., 2014; Rodriguez-Sanz et al., 2014a; Corredoira et al., 2017), indicating an epigenetic reprogramming after microspore induction to a totipotent state and embryogenesis initiation. More recently, it has been demonstrated that treatment with the DNA demethylating agent azacytidine reduces global DNA methylation of isolated microspore cultures and concomitantly increases embryogenesis initiation rates (Solis et al., 2015). In contrast to DNA methylation, much less is known about the dynamics and possible role of histone methylation marks in the process, except for our previous study (Rodriguez-Sanz et al., 2014b) that reported modifications of histone acetylation and methylation levels throughout microspore embryogenesis progression.

Histone methylation is a prominent epigenetic modification that is involved in the regulation of transcription and formation of heterochromatin, and it plays key roles in the regulation of numerous processes of development. This complex epigenetic mark can occur at different residues, lysine or arginine, in distinct sites of the protein sequence and can add different numbers of methyl groups. It has been reported that methylation of lysines is essential in different developmental processes, and this modification is very dynamic; levels of lysine methylation are maintained by the combined activity of writer enzymes (histone lysine methyltransferases) and eraser enzymes (histone demethylases), which add and remove methyl groups from lysines of histones. In plants, histone lysine methylation can occur at lysines of several positions in histones H3 and H4, mainly Lys4 (K4), Lys9 (K9), Lys27 (K27) and Lys36 (K36) of histone H3, and Lys20 (K20) of histone H4 (Naumann et al., 2005). Regarding histone H3 modifications, methylation at positions K9 and K27 is generally related to gene silencing while active genes are associated with methylation at K4 and K36. Among these epigenetic marks, H3K9 methylation is one of the most extensively studied modifications, with major functions in transcriptional control and heterochromatin maintenance (Jenuwein, 2006). In Arabidopsis, methylation of H3K9 is usually found in the mono (H3K9me) and dimethylated (H3K9me2) forms, with the trimethylated (H3K9me3) form being more scarce (Liu et al., 2010). Methylation of H3K9 has been related to gene inactivation in euchromatin and to heterochromatin (Pontvianne et al., 2010).

Due to the dynamic nature and potential reversibility of histone methylation marks, histone lysine methyl transferases (HKMTs) have attracted great interest in recent years as key epigenetic regulators of development. Several HKMT families have been identified in plants, and most are characterized by their homology with the animal and yeast SET domain (Liu et al., 2010). Polycomb repressive complexes (PRC) are well known epigenetic executors containing proteins with SET domains; in plants, PRC2 has been identified with histone methyltransferase activity specific for H3K27 (Köhler and Villar, 2008). Two plant HKMT families, the SUVHs and SUVRs proteins, mainly catalyze H3K9 methylation and mark inactive chromatin. In Arabidopsis, there are 10 SUVH and 4 SUVR genes (Baumbusch et al., 2001; Pontvianne et al., 2010). Among these enzymes, SUVR4 HKMT is closely related to the animal G9a HKMT, which has a dominant role in H3K9 methylation in early embryogenesis in mammals (Tachibana et al., 2002); SUVR4 HKMT has histone methyltransferase activity in plants and requires mono-methylated H3K9 as substrate (Thorstensen et al., 2006). Although less known than HKMTs, plant histone demethylases have also been reported as potential regulators of several developmental pathways (Luo et al., 2014; Prakash et al., 2014). Two main families of histone demethylases, lysine-specific demethylase 1 (LSD1) and Jumonji C (JmjC), homologous to the mammalian ones, have been identified in plants with activity on different histone residues (Liu et al., 2010; Luo et al., 2014; Prakash et al., 2014). In addition to H3K4 demethylation activity, LSD1 is also involved in removing H3K9 methylation in mammalian cells (Metzger et al., 2005; Liu et al., 2010; Prakash et al., 2014).

Since alterations in epigenetic marks have been implicated in many human diseases, including cancer, the targeting of histone methyltransferases and demethylases has recently become a key strategy in the latest developments for epigenetic drug discovery. In this regard, several inhibitors of histone methyltransferase enzymatic activities have been developed; they have been reported as novel therapeutic agents (Zagni et al., 2013; Pappano et al., 2015). Molecules with histone methyltransferase inhibition activity were first reported to induce reprogramming in different mammalian *in vitro* systems in which somatic cells were converted into pluripotent stem cells, for potential use in stem cell research and cell therapy applications (Shi et al., 2008a; Lin and Wu, 2015). The small molecule BIX-01294 is a diazepin-quinazolin-amine derivative which has been identified as a very specific inhibitor of G9a histone lysine methyltransferase and reduces bulk H3K9me2 levels in several mammalian cell lines; it can impair the closely related GLP HKMT only at very high concentrations (Kubicek et al., 2007). BIX-01294 has also been shown to improve the reprogramming efficiency of neural progenitor cells toward induced pluripotent stem cells (iPSCs) (Shi et al., 2008a,b) as well as to improve reprogramming in other somatic cell systems of mammals (Chen et al., 2015; Lin and Wu, 2015; Huang et al., 2016). However, BIX-01294 has not yet been tested in plant systems.

In the present work, we have analyzed changes in H3K9 methylation and the expression profiles of a histone methyltransferase of H3K9, *HKMT SUVR4-like*, and a histone demethylase, *LDS1-like*, during *in vitro* microspore reprogramming and embryogenesis initiation and progression in *B. napus* (rapeseed), in comparison with profiles during *in vivo* gametophytic development. We have also analyzed, the effects of treatments with the HKMT inhibitor BIX-01294, used for the first time in plants, on the efficiency of induction and progression of microspore embryogenesis, as well as its effects on bulk H3K9 methylation levels, DNA methylation and chromatin organization. SUVR4-like gene was selected for the analysis because of its expression in early embryogenesis and its homology with G9a of mammals. Moreover, to evaluate whether findings could be extended to other plant species and *in vitro* systems, H3K9 methylation analysis and BIX-01294 effects have also been analyzed in microspore embryogenesis cultures of the monocot *H. vulgare* (barley).

## Materials and Methods

### Plant Material and Microspore Embryogenesis *In Vitro* Cultures

As donor plants, rapeseed, *B. napus* L. cv. Topas seedlings were grown in a growth chamber (Sanyo, 14.000 lx, relative humidity 60%) under controlled conditions at 15°C day, 16 h photoperiod, and 10°C night, and used to obtain microspores and pollen grains developed *in vivo* for analyses of the gametophytic pathway, as well as for microspore *in vitro* cultures. For some experiments, winter barley, *H. vulgare* L. cv. Igri plants were also used. Barley seeds were vernalized in soil for 4 weeks at 4°C. After that, they were transferred to a plant growth chamber (Sanyo) (relative humidity about 70%) at 12°C with a 12 h photoperiod (10,000–16,000 lx) for 1 month, and then transferred to a greenhouse under a controlled temperature of 18°C.

Vacuolated microspores and tricellular pollen grains developed *in vivo* were isolated from anthers, in both plant species. *In vitro* cultures of isolated microspores and microspore embryogenesis induction were performed by stress treatments of 32°C in *B. napus* (Prem et al., 2012) and 4°C in *H. vulgare* (Rodríguez-Serrano et al., 2012), initiating the culture with vacuolated microspores, as the most responsive stage for embryogenesis induction in both species.

### Treatments of Microspore Cultures with BIX-01294

The histone methylation inhibitor BIX-01294 (Epigentek) was added to the microspore culture plates at the time of culture initiation at different concentrations, 0.5, 1, 2.5, and 5 μM, from a stock solution of 5 mg/ml (8.33 mM) in DMSO, keeping parallel plates without the drug as control (untreated cultures). Short BIX-01294 treatments were performed from culture initiation during 4–6 days, time point of the proembryo formation stage in both *in vitro* microspore cultures, rapeseed (Prem et al., 2012) and barley (Rodríguez-Serrano et al., 2012). Long BIX-01294 treatments were carried out in rapeseed from culture initiation until the stage of cotyledonary embryo formation (around 30 days).

Number of “proembryos” and “embryos” were quantified at defined time points of the microspore embryogenesis cultures. Randomly obtained micrographs from inverted microscope and stereomicroscope were collected from untreated and BIX-treated microspore culture plates. “Proembryos” were identified as rounded multicellular structures with higher size and density than microspores, still surrounded by the exine. Mean percentages of “proembryos” and total number of “embryos” (fully developed) per Petri dish was obtained from random samples of two independent experiments and different culture plates per each *in vitro* system. A minimum of 1000 proembryo/embryo structures were counted for each culture time point and treatment in each plant species. The results were shown in graphs in which columns represented mean values and bars represented standard error of the means. Significant differences were assessed by one-way ANOVA analysis of variance followed by Tukey’s multiple comparison test at *P* ≤ 0.05.

### Quantification of Global H3K9 Methylation

To compare levels of global H3K9 methylation of samples at different developmental stages and between untreated and BIX-treated samples, an EpiQuik Global Histone H3K9 Methylation Assay Kit (Colorimetric) (EpiGentek, Farmingdale, NY, United States) was used according to the manufacturer’s instruction. Hundred milligram of each sample were homogenized, nuclei and histone extraction were sequentially performed following the extraction procedure and solutions of the kit. Histone protein concentration was measured by the Bradford method, and the protein concentrations of histone extracts were adjusted in all samples to 300 ng/μl. In short, 1.5 μg histone proteins were spotted on the strip wells. Methylated histone H3K9 was detected with a specific antibody which was bound to a horseradish peroxidase-conjugated secondary antibody; amounts of methylated histone H3K9 were quantified by a color development reagent and were proportional to the intensity of color. Color density was measured by absorbance (optical density, OD) on the microplate reader at 450 nm and the amount of methylated H3K9 was proportional to the OD. Blanks and negative control (standard histone extract with no H3K9 methylation, provided in the assay kit) OD readings were subtracted to the sample OD readings. Assays were performed in triplicate. Results are presented as mean OD ± SE. Significant differences were tested by one-way ANOVA analysis of variance followed by Tukey’s multiple comparison test at *P* ≤ 0.05.

### Fixation and Low Temperature Processing for Microscopic Analysis

Freshly isolated microspores, pollen grains and *in vitro* samples from different culture times were collected and fixed overnight at 4°C with 4% paraformaldehyde in phosphate-buffered saline (PBS). After fixation, samples were processed for structural analysis and immunofluorescence. Fixed samples were washed in PBS, dehydrated through an acetone series (30, 50, 70, 90, and 100%) and embedded in Technovit 8100 resin (Kulzer, Germany) at 4°C. Resin polymerization was carried out at 4°C. The blocks were sectioned at 1–2 μm thickness and stained with 1% toluidine blue, mounted with Eukitt and observed under bright field microscopy, for structural analysis. Some sections were placed on aminopropyl-triethoxi-silane (APTES)-coated slides, and stored at 4°C; they were later used for immunofluorescence.

### Immunofluorescence and Confocal Analysis

Immunofluorescence was performed to localize 5-methyl-deoxy-cytidine (5mdC) and histone H3K9me2, essentially as previously described (Testillano et al., 2013; Rodriguez-Sanz et al., 2014b; Testillano and Risueno, 2016). Historesin semithin sections were first blocked with 5% Bovine Serum Albumin (BSA) in PBS for 10 min, and incubated for 1 h with anti-H3K9me2 rabbit polyclonal (Diagenode) and anti-5mdC mouse monoclonal (Eurogentec) antibodies diluted 1:50 in 1% BSA. In the case of 5mdC Immunofluorescence, prior to antibody incubations, DNA of sections was denaturated with 2N HCl for 45 min. After three rinsing steps in PBS, all sections were incubated with Alexa Fluor 488-labeled anti-rabbit or anti-mouse IgG antibody diluted 1:25 in 1% BSA, in the dark, for 45 min. After some washing in PBS, nuclei were stained with DAPI. After some final washing with PBS, sections were mounted in Mowiol and examined in a confocal microscope (Leica TCS-SP5-AOBS, Vienna, Austria). Confocal optical sections were collected at 0.2 μm z-intervals over a total thickness of 2 μm; images of maximum projections were obtained from z-stacks of 10 optical sections each with software of the confocal microscope (Leica software LCS version 2.5). For an accurate comparison among immunofluorescence signals of different developmental stages and treatments, confocal microscopy images were captured using the same laser excitation and sample emission settings in all immunofluorescence preparations of each species, rapeseed or barley.

Negative controls were performed avoiding the primary antibodies, anti-H3K9me2 or 5mdC. For 5mdC immunofluorescence, an additional control was performed by avoiding the denaturation step and replacing HCl by PBS.

Quantification of the H3K9me2 immunofluorescence intensity was performed with ImageJ software over confocal maximum projections, which were obtained as described above. Individual nuclei were outlined as ROIs (regions of interest) and fluorescence intensity values were obtained, in arbitrary units, for each developmental stage. Nuclei from 10 to 20 structures from three different immunofluorescence experiments and two biological replicates were measured per developmental stage. Significant differences were tested by one-way ANOVA analysis of variance followed by Tukey’s multiple comparison test at *P* ≤ 0.05.

For quantification of the 5mdC spots per nucleus, confocal images of maximum projections, obtained as described above, were used. 5mdC spots were counted in 80–100 nuclei per treatment, in randomly chosen images from two replicates. Results were classified in two categories: nuclei with 0 or 1 spots (nuclei with low DNA methylation) and nuclei with 2 or more spots (nuclei with high DNA methylation), and quantification was presented in histograms showing percentages of nuclei of each category in control and BIX-treated cultures.

### Quantitative Real-Time PCR (qRT-PCR)

Quantitative gene expression analyses were performed by qRT-PCR in *B. napus* freshly isolated vacuolated microspores and pollen grains from anthers, and *in vitro* embryogenesis samples from cultures at different time-points that correspond to the following developmental stages: proembryos (early stage after microspore reprogramming and embryogenesis initiation) and cotyledonary embryos (advanced stage of embryo differentiation). Total RNA from samples was isolated with the RNeasy^®^ Plant Minikit (Qiagen) according to the manufacturer’s instructions. As PCR templates, cDNA was generated from total RNA isolated from the different culture samples at the analyzed stages, using the Superscript^TM^ II reverse transcriptase enzyme (Invitrogen), according to Solis et al. (2012). Quantitative real-time PCR was performed using the SsoAdvanced^TM^ Universal SYBR^®^Green Supermix on the iQ^TM^5 Real-Time PCR Detection System (Biorad).

For the expression analysis of *BnHKMT SUVR4-like* gene, the oligonucleotides used were: 5′ TTGTTGCGTGAGCTGTAAGG 3′ and 3′GGGCAGTCTTGGCAGTAAAA 5′, from the sequence of the *HKMT* gene of *B. rapa* (Bra040197 accession number in *Brassica* gene database^1^), homologous of *SUVR4* HKMT gene of Arabidopsis (Thorstensen et al., 2006). For the expression analysis of *BnLSD1-like* gene, the oligonucleotides used were: 5′-GGAACTTGTCGATGGCGTAT-3′ and 3′-AGTGACGGGGTTGTGGTTTA-5′, from the *B. rapa LSD1-like* gene sequence (NCBI Reference Sequence: XM_009114794.1), which encodes a lysine-specific histone demethylase 1, homolog 1. Three technical replicates were performed for each qPCR reaction. Conditions of qPCR reaction were as follows: initial denaturation at 95°C for 30 s, followed by forty cycles of 5 s at 95°C and 30 s at 56°C. After each run, by heating the samples from 65 to 95°C a dissociation curve was acquired to check for amplification specificity. Serial dilutions of cDNA were used to determine the efficiency curve of each primer pair according to Solis et al. (2016). As internal reference gene, Actin II was used. Data was analyzed with the Bio-Rad CFX Manager 3.0 (3.0.1224.1015) (Biorad), using the Livak calculation method (Livak and Schmittgen, 2001). Transcript levels were normalized to vacuolated microspore stage levels. Differences among stages were tested by one-way ANOVA analysis of variance followed by Tukey’s multiple comparison test at *P* ≤ 0.05.

## Results

### Histone H3K9 Methylation Level and Distribution Pattern during Microspore Embryogenesis, in Comparison with Pollen Development

**FIGURE 1:**
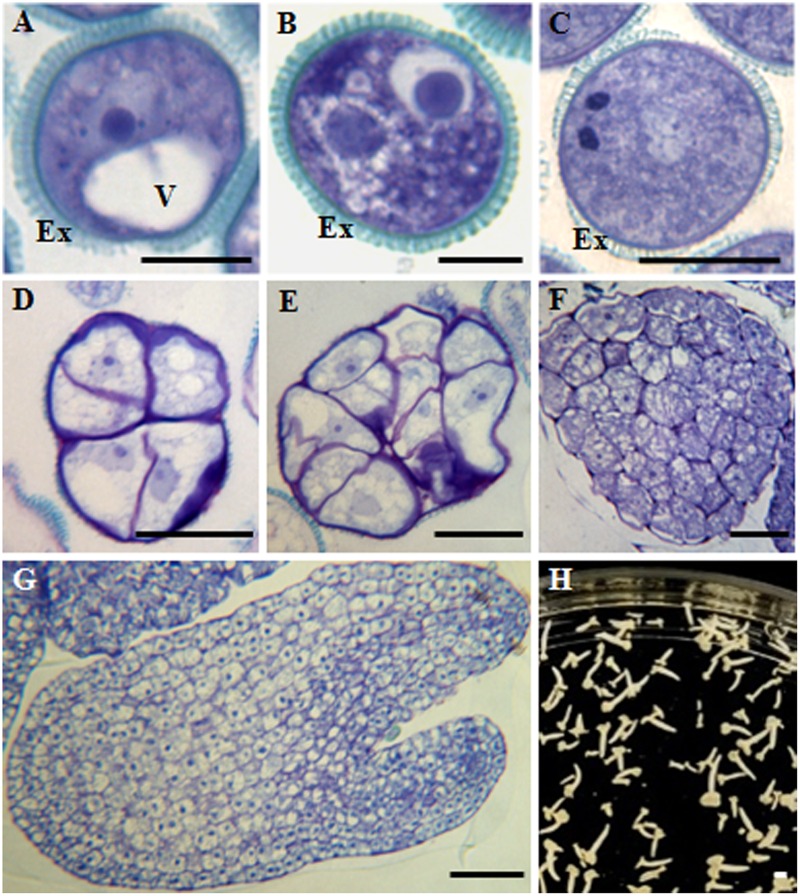
Main stages of pollen development and microspore embryogenesis of *Brassica napus*. Micrographs of semithin sections stained by Toluidine blue showing the cellular organization. **(A)** Vacuolated microspore. **(B,C)** Gametophytic development. **(B)** Bicellular pollen grain. **(C)** Tricellular pollen grain. **(D–H)** Microspore embryogenesis. **(D,E)** Proembryos. **(F)** Globular embryo. **(G)** Torpedo embryo. **(H)** Cotyledonary embryos, panoramic view of a Petri dish of a microspore embryogenesis culture after 30 days. Ex, exine; V, vacuole. Bars represent: **(A–C)**: 10 μm, **(D–F)**: 20 μm, **(G)**: 50 μm, **(H)**: 1 mm.

During *in vivo* anther development of *B. napus*, after meiosis, microspores developed to form the so-called vacuolated microspores (**Figure 1A**) which, after asymmetric division, produced the bicellular pollen grain; it contained the small generative cell inside the cytoplasm of the larger vegetative cell (**Figure 1B**). Following the gametophytic pathway, the generative cell divided and formed the two sperm cells, giving rise to the tricellular pollen grain (**Figure 1C**). At the responsive stage of the vacuolated microspore, by the application of a heat stress treatment *in vitro*, isolated microspores could be reprogrammed, initiating the embryogenesis pathway. The first embryogenic division of the vacuolated microspore was symmetric and gave rise to two-cell proembryos whose cells and nuclei were similar in size and organization. Then, cell proliferation increased and multicellular proembryos were formed (**Figures 1D,E**). As embryogenesis progressed, globular embryos were produced and later elongated to form heart-shaped and torpedo embryos (**Figures 1F,G**), which developed and differentiated, leading to the formation of cotyledonary embryo (**Figure 1H**).

To evaluate whether findings in rapeseed could be extended to other microspore embryogenesis systems, analyses were also performed in *H. vulgare*, barley, a monocot species in which microspore embryogenesis is induced by cold stress (Rodríguez-Serrano et al., 2012), instead of heat, as is the case for the dicot *B. napus*.

**FIGURE 2:**
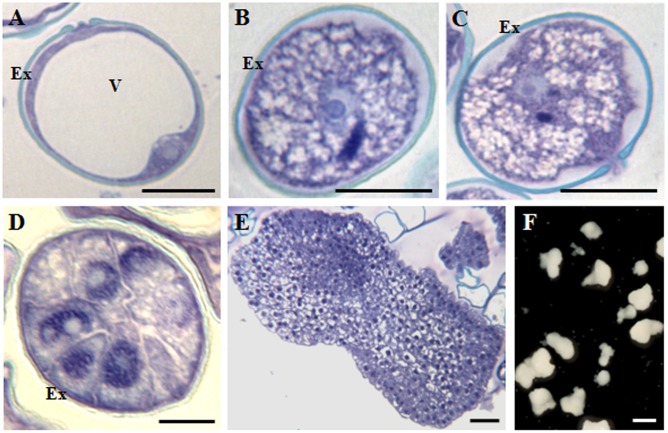
Main stages of pollen development and microspore embryogenesis of *Hordeum vulgare*. Micrographs of semithin sections stained by Toluidine blue showing the cellular organization. **(A)** Vacuolated microspore. **(B,C)** Gametophytic development. **(B)** Bicellular pollne. **(C)** Tricellular pollen. **(D–F)** Microspore embryogenesis. **(D)** Proembryo. **(E)** Transitional embryo. **(F)** Coleoptilar embryos, with different degrees of development, panoramic view of a Petri dish of a microspore embryogenesis culture after 30 days. Ex, exine; V, vacuole. Bars represent: **(A–C)**: 10 μm, **(D)**: 20 μm, **(E)**: 100 μm, **(F)**: 1 mm.

In barley, vacuolated microspores (**Figure 2A**) yielded *in vivo* bicellular (**Figure 2B**) and tricellular pollen grains (**Figure 2C**). After induction by cold stress, in isolated microspore *in vitro* cultures, responsive microspores reprogrammed, initiated the embryogenesis pathway and divided producing proembryos of several cells that appeared confined by the exine at early stages (**Figure 2D**). In subsequent stages, microspore embryogenesis progressed, the exine broke down and proembryos developed giving rise to globular, transitional (**Figure 2E**), scutellar, and coleoptilar monocot embryos (**Figure 2F**).

**FIGURE 3:**
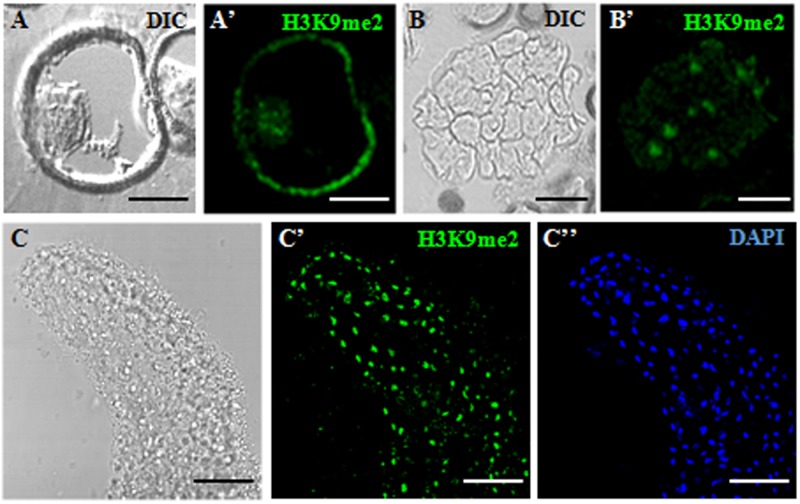
Immunofluorescence of H3K9me2 during microspore embryogenesis initiation and progression of *Brassica napus*. Confocal laser scanning microscopy analysis of vacuolated microspores **(A,A’)**, proembryos **(B,B’)** and cotyledonary embryos **(C–C”)**. **(A–C)**: Nomarsky’s differential interference contrast (DIC) images showing the cellular organization of the different structures. **(A’–C’)**: H3K9me2 immunofluorescence signal over nuclei (green). **(C”)**: DAPI staining of nuclei (blue) of a region of the cotyledon. The same structures are visualized under different microscopy modes in (**A,A’)**, **(B,B’)** and **(C–C”)**. The exine showed unspecific autofluorescence in some images **(A’)**. Bars represent: **(A,A’)**: 10 μm, **(B,B’)**: 20 μm, **(C–C”)**: 50 μm.

**FIGURE 4:**
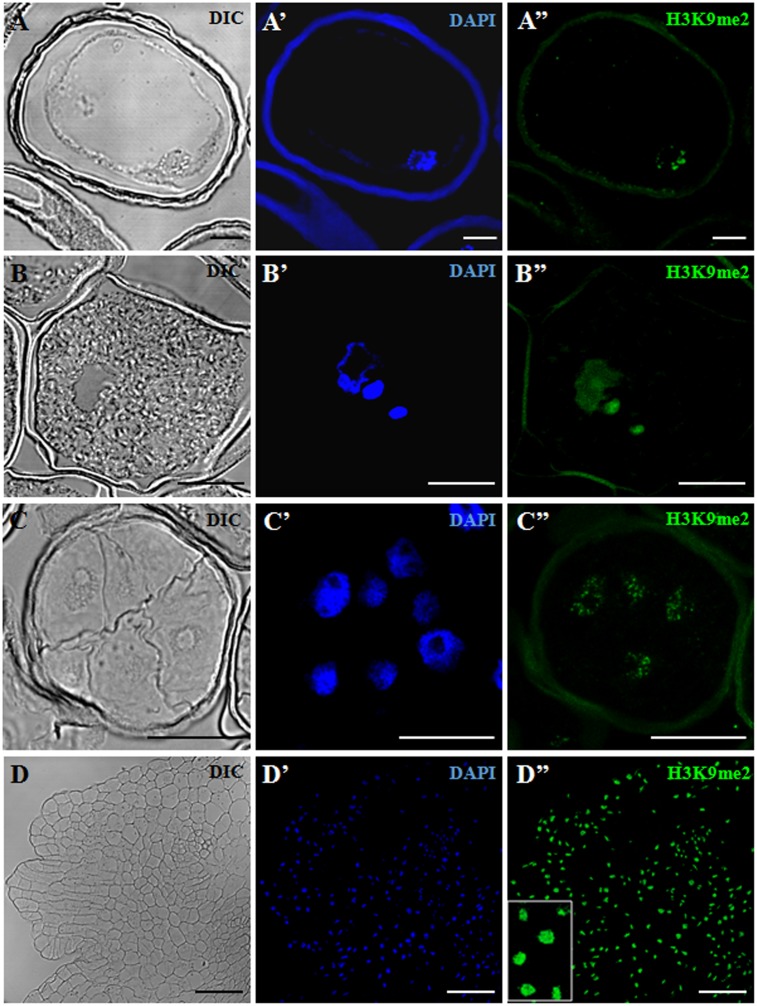
Immunofluorescence of H3K9me2 during pollen development and microspore embryogenesis of *Hordeum vulgare*. Confocal laser scanning microscopy analysis of vacuolated microspores, starting point of the two developmental pathways **(A–A”)**, tricellular pollen, advanced stage of gametophytic development **(B–B”)**, proembryos, early stage after reprogramming **(C–C”)** and coleoptilar embryo, advanced embryogenesis stage **(D–D”)**. **(A–D)**: Nomarsky’s differential interference contrast (DIC) images showing the cellular organization of the different structures. **(A’–D’)**: DAPI staining of nuclei (blue). **(A”–D”)**: H3K9me2 immunofluorescence signal over nuclei (green). The same structures are visualized under different microscopy modes in **(A–A”)**, **(B–B”)**, **(C–C”)** and **(D–D”)**. Inset shows a detail of **(D”)** at higher magnification. The exine showed unspecific autofluorescence in some images **(A’,A”,B”,C”)**. Bars represent: **(A–A”)**, **(B–B”)**: 10 μm, **(C–C”)**: 20 μm, **(D–D”)**: 75 μm.

Changes in H3K9 methylation levels and distribution patterns were analyzed during the gametophytic and embryogenic pathways, in the two species studied, rapeseed and barley. Immunofluorescence assays with specific H3K9me2 antibodies were performed to analyze the distribution of this epigenetic mark during the process. Image acquisition was carried out with confocal microscopy under the same excitation and emission settings for all immunofluorescence preparations. This procedure permitted an accurate comparison among signals of different developmental stages throughout the process (**Figures 3**, **4**). Maximum projections of z-stacks of 10 optical sections captured at 0.2 μm intervals were used to compare signal intensities among developmental stages. In *B. napus*, the vacuolated microspore showed immunofluorescence signal of mid intensity in the nucleus, which appeared in a peripheral location at this developmental stage (**Figures 3A,A’**). After embryogenesis induction, H3K9me2 immunofluorescence signals with a similar intensity to vacuolated microspore nuclei were found over the nuclei of proembryo cells (**Figures 3B,B’**). At advanced embryogenesis stages, H3K9me2 signal increased in globular, torpedo and cotyledonary embryos which all exhibited an intense immunofluorescence over cell nuclei (**Figures 3C–C”**). No significant labeling was observed in any other subcellular compartment at any stage, only the microspore wall, the exine, showed non-specific autofluorescence (**Figures 3A,A’**). Controls replacing the primary antibody with PBS did not show any labeling (data not shown).

H3K9me2 immunofluorescence assays were also performed in barley, in the following developmental stages of the two microspore pathways: vacuolated microspores (**Figure 4A**) as initial developmental stage, tricellular pollen (**Figure 4B**) as late stage of the gametophytic pathway, and two (early and advanced) stages of microspore embryogenesis, early proembryos (**Figure 4C**) and transitional embryos (**Figure 4D**). In barley cells, chromatin organization was found to be different to that of rapeseed; DAPI staining showed that chromatin was distributed throughout the entire nuclear area, in a dense chromatin pattern (**Figures 4A’–D’**), which is the typical chromatin organization of this species, reported for these cell types (El-Tantawy et al., 2014). Confocal microscopy analyses of the H3K9me2 immunofluorescence assays under the same excitation/emission settings for all samples, revealed faint H3K9me2 fluorescence signals in the vacuolated microspore nucleus, distributed as numerous, very small spots over the nucleus (**Figure 4A”**). In tricellular pollen, H3K9me2 labeling increased (**Figure 4B”**); immunofluorescence was very intense on the two sperm nuclei, covering the whole nuclear area; the vegetative nucleus, which exhibited a lobulated shape at this late developmental stage, showed less intense labeling homogenously distributed over the nucleus (**Figure 4B”**). After microspore reprogramming and embryogenesis induction, barley microspore proembryos exhibited large rounded nuclei with intense DAPI staining (**Figure 4C’**). Proembryo nuclei showed much lower H3K9me2 labeling than tricellular pollen, with immunofluorescence signal forming a very thin reticulum over the nuclei (**Figure 4C”**). At advanced embryogenesis stages, concomitantly with cell differentiation, in globular, transitional and coleoptilar embryos, H3K9me2 signal markedly increased over most nuclei which showed intense immunofluorescence (**Figure 4D”**). Control experiments without the primary antibody did not provide any signal.

**FIGURE 5:**
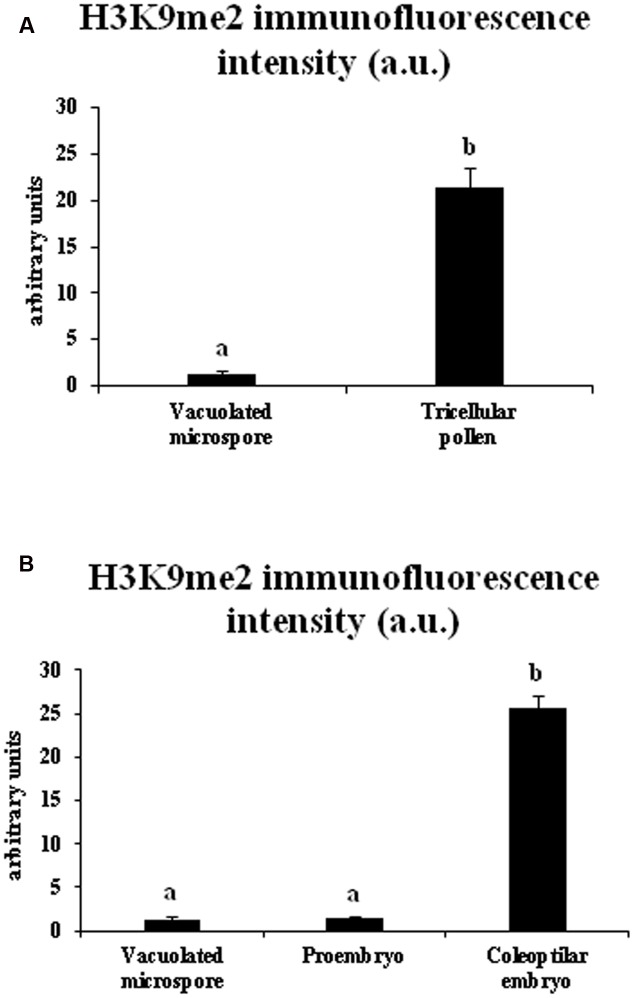
Quantification of H3K9me2 immunofluorescence signal intensity during pollen development and microspore embryogenesis in *Hordeum vulgare.* Histograms represent the level of immunofluorescence intensity, in arbitrary units, as measured by ImageJ software tools over confocal maximum projections images, in different stages of the two microspore pathways, gametophytic development (**A**: vacuolated microspore and tricellular pollen), and microspore embryogenesis (**B**: vacuolated microspore, proembryo and coleoptilar embryo). Columns represent mean fluorescence intensity (±SEM). Different letters indicate significant differences according to ANOVA and Tukey’s test at *p* ≤ 0.05.

Quantification of the intensity of H3K9me2 immunofluorescence signals was performed using appropriate image analysis software tools (see Materials and Methods) in cell nuclei at different developmental stages during gametophytic and embryogenic pathways, in barley. The results made it possible to quantitatively assess the immunofluorescence assays, supporting the variations of H3K9me2 observed by confocal microscopy. Quantitative image analysis showed a highly significant increase in H3K9me2 signal during pollen development, with low signal intensity in vacuolated microspore nuclei (1.27 ± 0.44 fluorescence arbitrary units) and much higher signal in sperm nuclei of tricellular pollen (21.40 ± 2.05 fluorescence a.u.) (**Figure 5A**). After induction of microspore reprogramming and embryogenesis initiation, proembryo nuclei showed a low value of H3K9me2 immunofluorescence intensity (1.49 ± 0.13 fluorescence a.u.), a value that was not significantly different from the immunofluorescence intensity of the vacuolated microspore (**Figure 5B**). At later developmental stages, nuclei of coleoptilar embryos exhibited very high immunofluorescence intensity values (25.56 ± 1.41 fluorescence a.u.) (**Figure 5B**).

**FIGURE 6:**
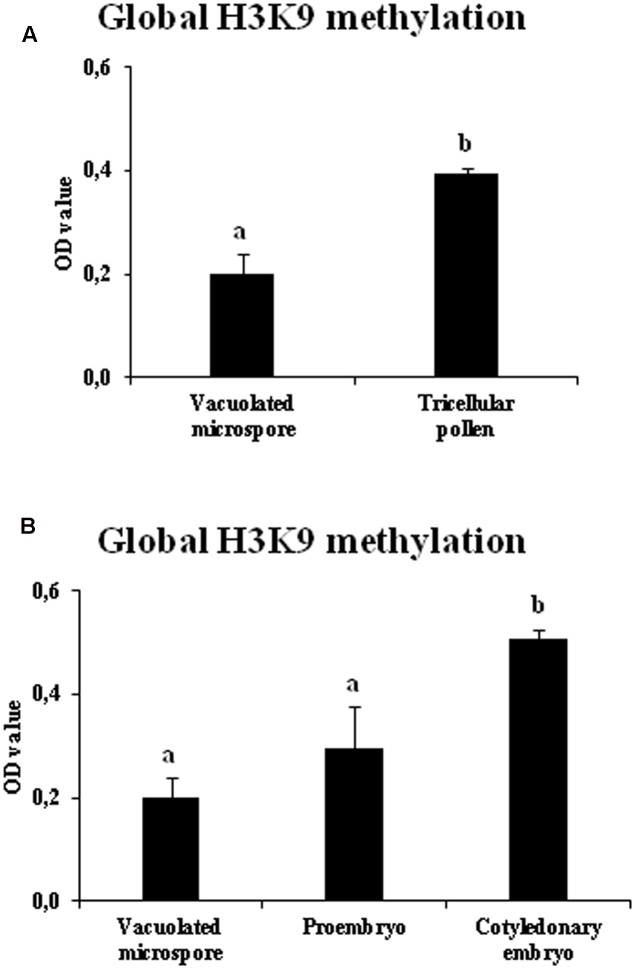
Quantification of global H3K9 methylation during pollen development and microspore embryogenesis of *Brassica napus*. Histograms represent the amount of H3K9 methylation, in optical density (OD) units at 450 nm (see Materials and Methods), in different stages of the two microspore pathways, gametophytic development (**A**: vacuolated microspore and tricellular pollen), and microspore embryogenesis (**B**: vacuolated microspore, proembryo and cotyledonary embryo). Columns represent mean optical density (OD) units at 450 nm (±SEM). Different letters indicate significant differences according to ANOVA and Tukey’s test at *p* ≤ 0.05.

Changes in the bulk H3K9 methylation levels were analyzed during the gametophytic and embryogenic pathways in rapeseed. Global H3K9 methylation levels were quantified in vacuolated microspores (initial stage for both pathways), tricellular pollen grains (late stage of the gametophytic pathway), proembryos (early stage after microspore reprogramming and embryogenesis initiation) and cotyledonary embryos (late stage of microspore embryogenesis). In the gametophytic pathway, results showed a significantly higher degree of histone H3K9 methylation in tricellular pollen in comparison with vacuolated microspore (**Figure 6A**). By contrast, in the embryogenesis pathway, early proembryos formed after microspore reprogramming exhibited similar low levels of H3K9 methylation to levels observed with vacuolated microspores (**Figure 6B**), whereas at advanced embryogenesis stages, with embryo differentiation, H3K9 methylation significantly increased in cotyledonary embryos (**Figure 6B**).

In short, the results showed that H3K9 methylation increased markedly with the progression of the gametophytic program while, after induction by stress, microspore embryogenesis initiation showed lower levels of H3K9 methylation, increasing at later embryogenesis stages, as embryo differentiation proceeded. Comparison of the two developmental programs, gametophytic and embryogenic, showed changes in bulk H3K9 methylation associated with the change in developmental program. These profiles were found in the two plant species, monocot and dicot, in which the change of developmental program was induced by different stress treatments (hot temperature in rapeseed and cold temperature in barley).

### Gene Expression Patterns of Histone Methyltransferase *BnHKMT SUVR4-Like* and Histone Demethylase *BnLSD1-Like* during Microspore Embryogenesis, in Comparison with Pollen Development

**FIGURE 7:**
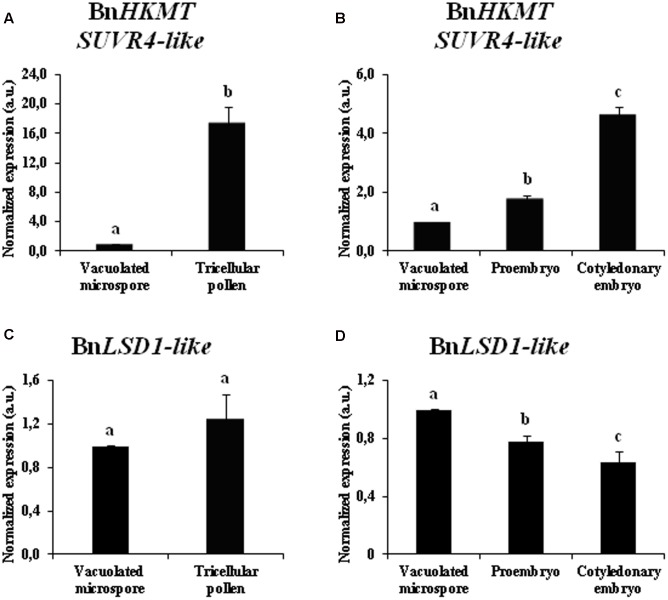
Gene expression patterns of *BnHKMT SUVR4-like* histone methyltransferase and *BnLSD1-like* demethylase during pollen development and microspore embryogenesis of *Brassica napus*, by quantitative qPCR. Histograms express relative changes of expression at different stages of gametophytic development **(A,C)** and microspore embryogenesis **(B,D)**: vacuolated microspore (starting point of the two pathways, before reprogramming), tricellular pollen (gametophytic pathway), proembryo and cotyledonary embryo (embryogenic pathway). Transcript levels were normalized to vacuolated microspore levels. Bars indicate the SEM. Different letters on columns indicate significant differences according to ANOVA and Tukey’s tests at *p* < 0.05.

During the two microspore developmental pathways, gene expression patterns of two enzymes that modify the methylation of H3K9, histone methyl transferase *BnHKMT SUVR4-like* and histone demethylase *BnLSD1-like*, were analyzed at different stages of microspore embryogenesis and pollen development of *B. napus. BnHKMT* gene is homologous to the *HKMT SUVR4* gene of Arabidopsis which encodes a HKMT with preference for H3K9me1 as substrate (Thorstensen et al., 2006). *BnLSD-like* is a LSD1, which removes methyl groups in mono and dimethylated forms from H3K4 and H3K9 (Metzger et al., 2005; Jiang et al., 2007; Liu et al., 2010). The qPCR results showed that *BnHKMT SUVR4-like* expression was highly induced during pollen maturation, with expression levels in tricellular pollen more than 15-fold higher than in vacuolated microspores (**Figure 7A**). In contrast, after microspore reprogramming and embryogenesis initiation, early proembryos showed moderate expression values, slightly higher than vacuolated microspores (**Figure 7B**). At later developmental stages, with microspore embryogenesis progression, *BnHKMT SUVR4-like* was up-regulated, with high expression levels in cotyledonary embryos (**Figure 7B**).

The gene expression analysis of the demethylase *BnLSD1-like* during the gametophytic pathway showed similar levels of expression in vacuolated microspores and tricellular pollen (**Figure 7C**). After microspore reprogramming, in the embryogenesis pathway, *BnLSD1-like* was progressively down-regulated, reaching the lowest value at advanced developmental stages, in cotyledonary embryos (**Figure 7D**). During microspore embryogenesis progression, the two genes displayed opposite expression patterns, *BnHKMT SUVR4-like* was up-regulated while *BnLSD1-like* was down-regulated at late embryo differentiation stages (**Figures 7B,D**).

The results indicated that histone methyl transferase *BnHKMT SUVR4-like* showed different expression patterns during gametophytic development and microspore embryogenesis initiation, patterns that in part correlated with global H3K9 methylation profiles. Expression of histone demethylase *BnLSD1-like* correlated with H3K9 methylation levels only during the microspore embryogenesis pathway.

### Effects of BIX-01294 Short Treatment on Microspore Embryogenesis Initiation and Histone H3K9 Methylation Level

To analyze the possible involvement of histone methylation in microspore embryogenesis, microspore cultures were treated with the inhibitor BIX-01294 (BIX) at different concentrations (1, 2.5, and 5 μM), and their effects on embryogenesis initiation (short-term treatment) and embryo formation/differentiation (long-term treatment) were evaluated.

**FIGURE 8:**
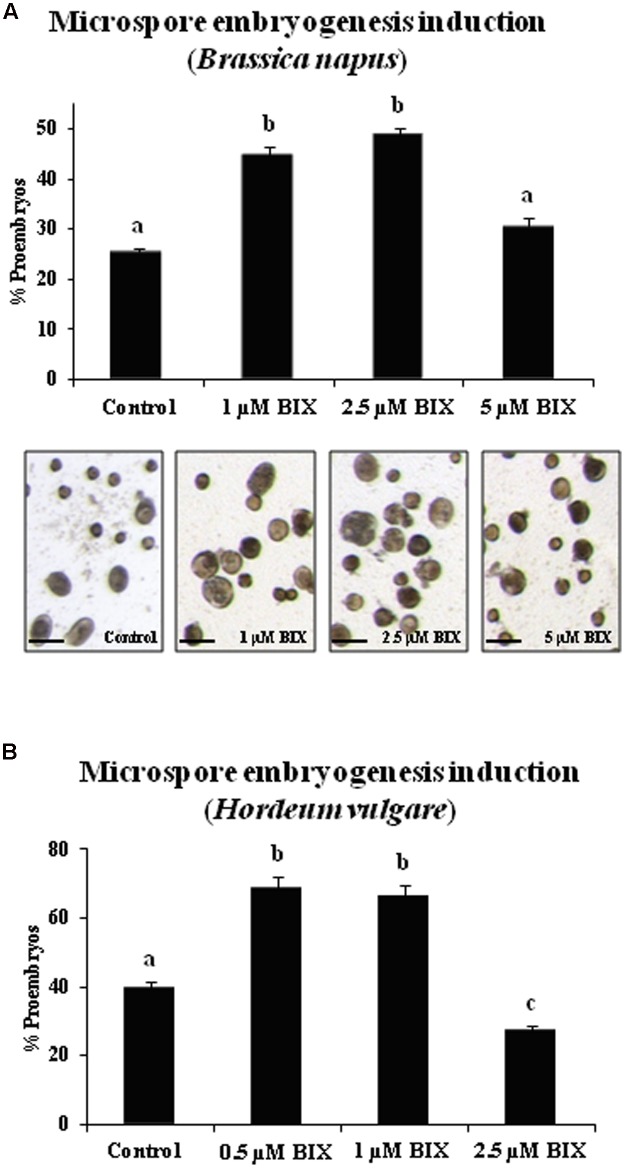
Effects of BIX-01294 short treatments on microspore embryogenesis induction of *Brassica napus* and *Hordeum vulgare*.**(A)**: Quantification of microspore embryogenesis induction and representative micrographs of control and BIX-treated cultures of *Brassica napus*.**(B)**: Quantification of microspore embryogenesis induction in control and BIX-treated cultures of *Hordeum vulgare*. Histograms show percentages of proembryos formed in microspore cultures untreated (control) and after short treatment (4 days) with BIX-01294 at different concentrations (1, 2.5, and 5 μM in *B. napus*, 0.5, 1, and 2.5 μM in *H. vulgare*); bars in columns indicate the SEM; different letters on columns indicate significant differences according to ANOVA and Tukey’s tests at *p* < 0.05. Micrographs show proembryos (larger, dense-rounded structures) in representative areas of control and BIX-treated cultures at 1, 2.5, and 5 μM concentrations, in rapeseed. Bars represent 50 μm.

For short treatments, BIX-01294 was added to rapeseed culture medium from the culture initiation stage until the proembryo formation stage (4 days). Proembryos formed at this culture stage could be clearly distinguished from the non-responsive microspores that were also present in the culture, since the proembryos were rounded structures that were larger and denser than microspores (**Figure 8A**). The quantification of the proembryos, at the same culture time point in untreated and BIX-treated microspore cultures, showed a significant increase in the proportion of proembryos in cultures treated with 1 and 2.5 μM BIX compared to control cultures (**Figure 8A**); this indicated that BIX treatment promoted embryogenesis initiation. Treatment with higher concentrations, such as 5 μM, reduced the proportion of proembryos (**Figure 8A**).

The effects of BIX-mediated inhibition of H3K9 methylation on microspore embryogenesis initiation were also evaluated in barley cultures. Short-term BIX-01294 treatments were performed by adding the drug to the medium from the initiation of the culture until the stage of proembryo formation (4 days), using the same concentrations as in rapeseed microspore cultures, 1 and 2.5 μM. A lower concentration, 0.5 μM, was also tested since the exine (microspore wall) of barley is much thinner than in rapeseed and would potentially facilitate penetration of drugs *in vivo*. The quantification of proembryos formed revealed higher proportions of proembryos in microspore cultures treated with 0.5 and 1 μM BIX than in untreated cultures, approximately a twofold increase for both concentrations (**Figure 8B**). BIX treatments with 2.5 μM concentration did not show significant effects on embryogenesis induction and produced a similar proportion of proembryos than untreated cultures (**Figure 8B**), indicating that in barley lower BIX-01294 concentrations were required to promote proembryo formation than in rapeseed.

**FIGURE 9:**
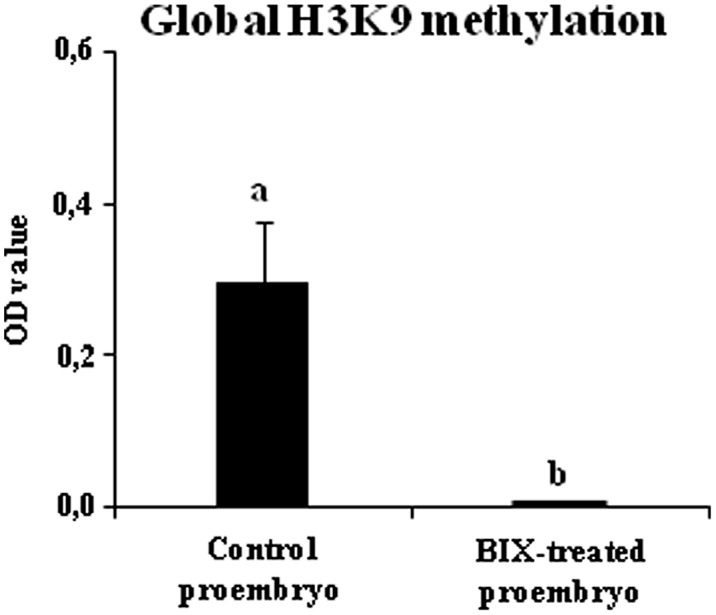
Effects of BIX-01294 short treatments on H3K9 methylation levels of proembryos of *Brassica napus*. Quantification of global H3K9 methylation levels, represented as mean optical density (OD) units at 450 nm (±SEM), in control (untreated) and 2.5 μM BIX-treated proembryos of *Brassica napus*, after 4 days in culture. Different letters on columns indicate significant differences according to ANOVA and Tukey’s tests at *p* < 0.05.

To evaluate whether BIX treatment had an inhibitory effect on histone methylation in microspore embryogenesis cultures, as reported for mammalian cells (Kubicek et al., 2007), the levels of H3K9 methylation were analyzed in proembryos that had been forming in control and 2.5 μM BIX-treated cultures of *B. napus* microspores for 4 days. The results showed that, in comparison with control cultures, BIX-treated proembryos showed a large decrease in H3K9 methylation which was below the sensitivity of the method used and could not be detected (**Figure 9**). This indicated that the compound significantly reduced histone H3K9 methylation during microspore embryogenesis initiation.

These results showed that short-term BIX-01294 treatment increased the proportion of microspores that initiated embryogenesis whereas it reduced H3K9 methylation.

### Effects of BIX-01294 Long Treatment on Microspore Embryogenesis Progression and Histone H3K9 Methylation Level

**FIGURE 10:**
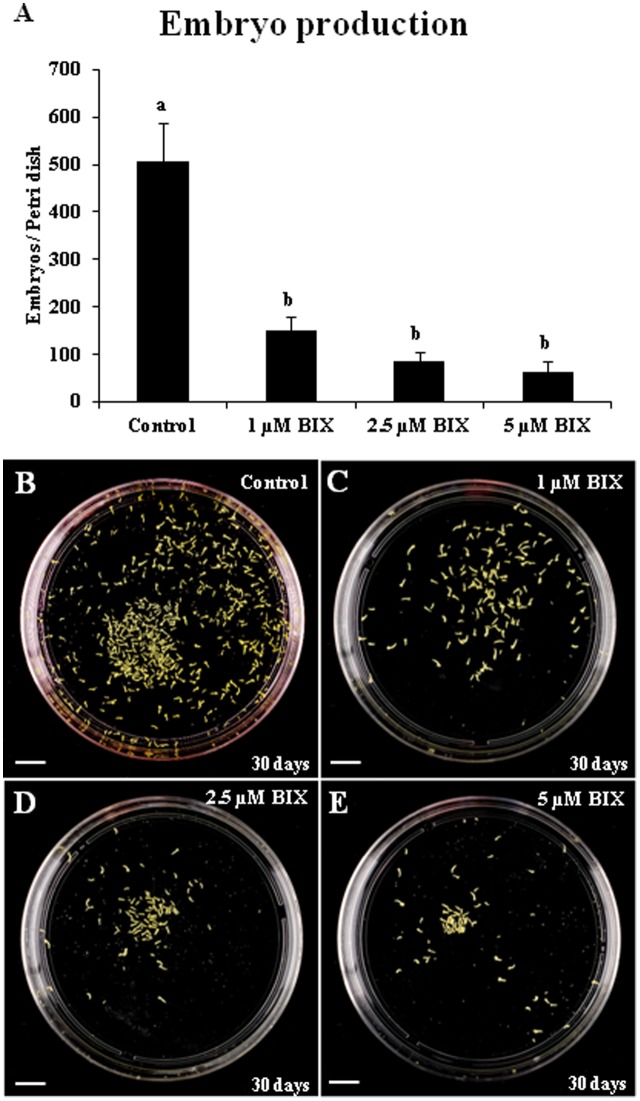
Effects of BIX-01294 long treatments on microspore-derived embryo production of *Brassica napus.* **(A)**: Quantification of the embryo production in control and BIX-treated cultures at 1, 2.5, and 5 μM concentrations. Columns represent mean values (±SEM) of the total number of embryos per Petri dish. Different letters on columns indicate significant differences according to ANOVA and Tukey’s tests at *p* < 0.05. **(B–E)**: Representative plates showing the microspore-derived embryos produced in control **(B)** and BIX-treated cultures at 1 μM **(C)**, 2.5 μM **(D)** and 5 μM **(E)** concentrations, after 30 days. Bars represent 10 mm.

Long treatments with BIX-01294 were performed to evaluate the effect of the drug on embryo production. These treatments were carried out in rapeseed microspore cultures for a period of 30 days from culture initiation, the time required for the embryo to complete development and reach the cotyledonary embryo stage. Parallel cultures were performed in the presence and absence of the drug and the production of fully developed embryos was quantified (**Figure 10**). The results showed a very marked reduction of embryo formation in BIX-treated cultures with all concentrations used, 1, 2.5, and 5 μM (**Figures 10A–E**), in comparison with untreated cultures (**Figure 10B**). This indicated that BIX treatment impaired further embryo development.

The inhibitory effect of BIX-01294 on histone methylation during long treatments was also evaluated by quantification of the levels of H3K9 methylation in 20-day-old embryos developed in untreated cultures and cultures treated with 1 μM BIX-01294, the lowest concentration that had a positive effect on embryogenesis induction in *B. napus* microspore cultures.

**FIGURE 11:**
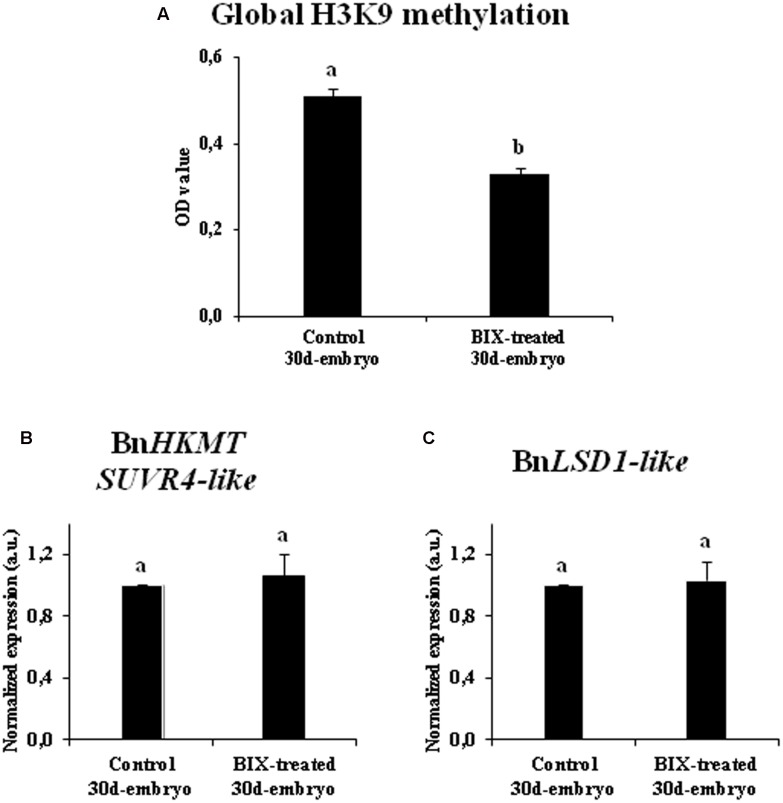
Effects of BIX-01294 long treatments on H3K9 methylation levels and gene expression of *BnHKTM SUVR4-like* histone methyltransferase and *BnLSD1-like* demethylase. **(A)**: Quantification of global H3K9 methylation levels, represented as mean optical density (OD) units at 450 nm (±SEM), in control and 2.5 μM BIX-treated embryos of *Brassica napus*, after 30 days in culture. **(B,C)**: Relative expression of histone methyltransferase *BnHKTM SUVR4-like* **(B)** and demethylase *BnLSD1-like* **(C)** in control and 2.5 μM BIX-treated embryos, after 30 days in culture. Transcript levels were normalized to control embryo levels. Bars indicate the SEM. Different letters on columns indicate significant differences according to ANOVA and Tukey’s tests at *p* < 0.05.

The comparative analysis showed a significant reduction of bulk H3K9 methylation level in microspore embryos treated with BIX-01294, in comparison with control embryos (**Figure 11A**). The gene expression of histone methyltransferase *BnHKMT SUVR4-like* and histone demethylase *BnLSD1-like* were also analyzed in both types of embryos. For these two genes, the qPCR analysis revealed no significant changes in expression between embryos treated with BIX-01294 and embryos developed in the absence of the drug (**Figures 11B,C**). These findings suggested that BIX-01294 had an inhibitory effect on histone H3K9 methylation in developing microspore embryos, reducing global H3K9 methylation levels, perhaps by affecting the methylation process and/or the enzymatic activity (similar to its reported activity in animal cells), rather than influencing the expression of methyltransferases and demethylases.

In short, these results indicated that long-term BIX-01294 treatment impaired embryo differentiation and reduced H3K9 methylation levels in developing embryos.

### Effects of BIX-01294 Treatment on Global DNA Methylation Distribution Patterns in Microspore Embryogenesis

We also evaluated the effect of BIX-01294 on DNA methylation in microspore embryogenesis cultures of rapeseed. Immunofluorescence assays with 5-methyl-deoxi-cytidine (5mdC) antibodies and confocal analysis were performed to study global DNA methylation nuclear distribution patterns of proembryos at early microspore embryogenesis stages, in BIX-treated cultures (1 and 2.5 μM concentrations), and untreated cultures.

**FIGURE 12:**
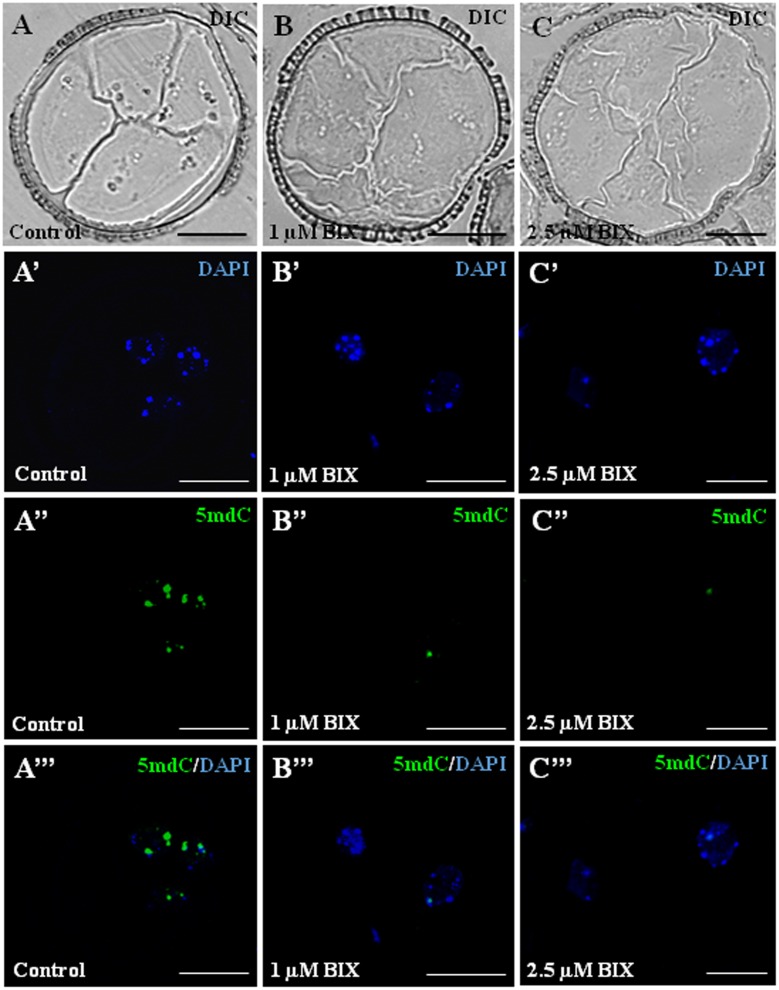
Distribution patterns of methylated DNA (5mdC) in microspore proembryos formed in control conditions and short BIX-01294 treatments. 5mdC immunofluorescence and confocal laser scanning microscopy analysis in *Brassica napus*. Microspore proembryos of control **(A–A”’)**, 1 μM **(B–B”’)** and 2.5 μM **(C–C”’)** BIX-01294 treated cultures. **(A–C)**: Nomarsky’s differential interference contrast (DIC) images of the proembryo structure. **(A’–C’)**: DAPI staining of nuclei (blue). **(A”–C”)**: 5mdC immunofluorescence signal (green). **(A”’–C”’)**: Merged images of DAPI (blue) and 5mdC immunofluorescence (green). The same structures are visualized under different microscopy modes in **(A–A”’)**, **(B–B”’)** and **(C–C”’)**. Bars represent 20 μm.

Proembryos formed after microspore reprogramming in cultures treated with 1 and 2.5 μM BIX exhibited a cellular organization similar to proembryos developed in control cultures (**Figures 12A–C**). Early proembryos were still surrounded by the microspore wall, the exine, and were formed by a few cells that exhibited clear cytoplasms, with some small vacuoles and a few starch deposits, and one relatively large, rounded nucleus which appeared clear under phase contrast microscopy (**Figures 12A–C**). DAPI-specific staining of DNA showed some brightly stained heterochromatin foci of variable size, mainly located at the nuclear periphery and dispersed within the euchromatin, which exhibited much lower DAPI-fluorescence (**Figures 12A’–C’**); DAPI fluorescence reflected the low-mid chromatin condensation pattern typical of *B. napus*, reported for these cell types (Seguí-Simarro et al., 2011). In untreated proembryos, 5mdC immunofluorescence signal was concentrated in several bright foci, mostly preferentially associated with heterochromatin masses (condensed chromatin masses) and localized at the nuclear periphery, as revealed by DAPI (**Figures 12A’–A”’**). After BIX treatment, 5mdC immunofluorescence labeling was much less intense than in untreated samples; the decrease in 5mdC signal was found with both concentrations, 1 and 2.5 μM. BIX-treated proembryo nuclei exhibited no or very low signal, and the nuclear pattern of 5mdC distribution was mainly concentrated in only 1 or very few small spots per nucleus (**Figures 12B’–B”’,C’–C”’**). Controls of immunofluorescence assays without the DNA denaturation step or eliminating the first antibody did not provide labeling in any sample (data not shown).

**FIGURE 13:**
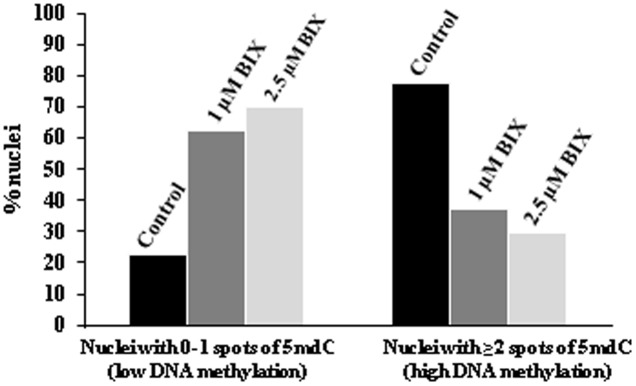
Quantification of effects of BIX-01294 on DNA methylation (5mdC) nuclear distribution patterns in microspore proembryos. Quantification of the percentage of nuclei showing: (a) 0 or 1 spot of 5mdC per nucleus (low DNA methylation), and (b) 2 or more spots of 5mdC per nucleus (high DNA methylation) in control, 1 and 2.5 μM BIX-treated proembryos of *Brassica napus.*

To assess the reduction of 5mdC labeling in BIX-treated proembryo nuclei, quantification of the number of 5mdC spots per nuclei in each sample was performed. The results revealed that most nuclei (80%) of untreated proembryos showed 2 or more 5mdC foci per nucleus (high DNA methylation) and only a small proportion of them showed no spots or only 1 (**Figure 13**). By contrast, in BIX-treated proembryos, the majority of nuclei exhibited 0 to 1 spots of 5mdC (low DNA methylation), and this proportion was higher with 2.5 μM than with 1 μM treatment (**Figure 13**).

The results indicated that the reduction of H3K9 methylation produced by short-term BIX-01294 treatment, was also accompanied by a decrease in global DNA methylation.

## Discussion

### Microspore Reprogramming, Totipotency and Embryogenesis Initiation Involve a Reduction in H3K9 Methylation, Whereas This Epigenetic Modification Increases Markedly in the Gametophytic Program

Several reports have shown in different plant systems that somatic embryogenesis initiation was associated with changes in chromatin structure leading to a less condensed state (Testillano et al., 2000, 2005; Seguí-Simarro et al., 2011; Feher, 2015). Chromatin modification and remodeling has also been reported during early zygotic embryogenesis and seed development (Baroux et al., 2007). It is also known that post-translational histone modifications, together with DNA methylation, contribute to driving changes in chromatin conformation and condensation (Yang et al., 2010; Eichten et al., 2014). A recent genome-wide analysis revealed the relationship between histone methylation regulation and reprogramming of gene expression in the vegetative to reproductive transition in the rice inflorescence meristem (Liu et al., 2015). In pollen, increasing evidence indicates epigenetic reprogramming through DNA methylation, histone methylation and siRNAs (Slotkin et al., 2009; Calarco et al., 2012; Solis et al., 2012; El-Tantawy et al., 2014). H3K9 methylation is one of the major histone modifications with central roles in the epigenetic control of many developmental processes. The analysis performed here revealed changes in bulk H3K9 methylation and its nuclear distribution, that are associated with the change in developmental program of the microspore. Progression of the gametophytic program produced a large increase of global H3K9 methylation levels. However, after *in vitro* induction by stress, microspore reprogramming, totipotency acquisition and embryogenesis initiation involved lower levels of H3K9 methylation, revealing an epigenetic change associated with the change in developmental program.

In plant genomes, H3K9 methylation is associated with DNA methylation and small RNAs, which are both essential drivers for the heterochromatin formation (Saze et al., 2012). In this regard, microspore embryogenesis initiation has also been associated with global DNA hypomethylation and with increasing histone acetylation, in several plant species (Solis et al., 2012; El-Tantawy et al., 2014; Li et al., 2014; Rodriguez-Sanz et al., 2014a,b; Feher, 2015). However, much less is known about histone methylation. H3K27 methyltransferases of the POLYCOMB REPRESSIVE COMPLEX 2 (PRC2) have been associated with prevention of pluripotency during differentiation or during somatic-to-reproductive cell fate transition, as well as with repression of lateral root formation in Arabidopsis (She et al., 2013; Gentry and Hennig, 2014; Gu et al., 2014; Feher, 2015). In the present work, low H3K9 methylation has been found to be associated with microspore reprogramming, totipotency acquisition and embryogenesis initiation, together with reduced DNA methylation. These epigenetic changes could lead to an increase of the cellular plasticity by promoting open chromatin states and facilitating the access of transcription factors to the chromatin fiber, especially those driving the change of developmental program.

Chromatin-modifying enzymes that affect the genome-wide distribution of histone marks have been proposed as modulators of cell reprogramming (Feher, 2015). Our results showed that the H3K9 methyltransferase gene *BnHKMT SUVR4-like* was expressed in *B. napus* microspores, pollen grains and embryos, with expression patterns that in part correlated with global H3K9 methylation profiles during gametophytic development and microspore embryogenesis. Although the activity of many other HKMT genes could also be involved, our findings suggest the contribution of this enzyme in the regulation of H3K9 methylation levels during both microspore developmental programs. By contrast, the expression results of the histone demethylase *BnLSD1-like* indicate that the participation of LSD1-like activities would not be very relevant in these microspore pathways in which other demethylases may act.

Previous studies in Arabidopsis mutants, of KRIPTONITE (KYP) histone H3K9 methyltransferase and H3K4 demethylase JMJ14, suggested that both activities were involved in the promotion of dedifferentiation and *in vitro* shoot organogenesis, through increased WUS expression (Grafi et al., 2011a; Li et al., 2011). Our results indicate that epigenetic control through H3K9 methylation may play a relevant role in both cell reprogramming and cell differentiation during microspore embryogenesis of *B. napus*. The decrease in global H3K9 methylation would allow the reprogramming of cells whereas the high expression, and presumably high activity, of histone lysine methyltransferases leading to increased H3K9 methylation levels could be considered as an impediment for cell reprogramming, as occurs in differentiating pollen grains.

### BIX-01294 Promotes Microspore Reprogramming, Totipotency and Embryogenesis Initiation while Inhibiting H3K9 Methylation

The small molecule BIX-01294 has been identified as a specific inhibitor for the histone methyltransferase G9a in mammalian cells, and its capacity to reduce H3K9me2 levels has been demonstrated *in vitro* (Tachibana et al., 2002; Kubicek et al., 2007). However, to our knowledge, it had never been tested in plant cells prior to the present work. Since our results showed that the change of developmental program of the microspore was associated with reduced levels of H3K9 methylation, we have tested the effects of BIX-01294 on the efficiency of microspore reprogramming to embryogenesis. The results showed a significant increase in microspore embryogenesis induction with short-term BIX-01294 *in vitro* treatments, together with a decrease in H3K9 methylation levels. In mammals, H3K9 methylation by G9a HKMT has been reported to lead to heterochromatinization, a mechanism of epigenetic silencing of embryonic genes (Feldman et al., 2006; Chen et al., 2015). Interestingly, the pharmacological inhibition of G9a HKMT-mediated H3K9me2 by BIX-01294 was reported to improve cell reprogramming and to enhance the generation of iPSCs from neuronal progenitor cells and mouse embryonic fibroblasts transduced with Oct/Klf4 (Shi et al., 2008a). This suggests that H3K9 methylation would interfere with, or be a barrier for, the reprogramming of somatic cells into iPSCs (Chen et al., 2015). Our results in BIX-treated microspore cultures would agree with an analogous effect of this small compound in promoting plant cell reprogramming to embryogenesis, associated with the inhibition of H3K9 methylation.

While it is well established that BIX-01294 has a high specificity for the inhibition of the histone methyl transferase G9a in mammals and therefore for the reduction of H3K9 methylation (Kubicek et al., 2007), no data is available on the action of this compound in plants. However, results presented here show a similar effect of the drug in plants, i.e., the reduction of H3K9 methylation. This effect, together with expression results of *BnHKMT SUVR4-like*, the close relation of this plant HKMT with the mammalian G9a (Liu et al., 2010), and the fact that both enzymes show a dominant role in H3K9 methylation, suggest that the functional specificity of BIX-01294 in plants and mammals may be analogous, although further work would be required to determine the chemical activity of BIX-01294 in plants.

In Arabidopsis, like in animals, increasing evidence indicates that H3K9me2 controls DNA methylation and vice versa (Soppe et al., 2002; Feng and Jacobsen, 2011; Du et al., 2012; Saze et al., 2012; Eichten et al., 2014). H3K9 methylation is associated with DNA methylation, and both epigenetic marks are essential for the formation of heterochromatin (Saze et al., 2012). Several heterochromatin-specific histone marks, like H4K20 and H3K27 methylation, can also affect DNA methylation (Naumann et al., 2005). Epigenetic inhibitors have been reported to affect chromatin condensation states and to influence cell proliferation and differentiation during plant development (Yang et al., 2010; Tokuji et al., 2011; Fraga et al., 2012; Solis et al., 2012). After microspore reprogramming, in microspore-derived proembryos of several species, a decondensed pattern of chromatin has been reported, as a characteristic feature of proliferating cells (Testillano et al., 2000; Bárány et al., 2005; Testillano et al., 2005; Seguí-Simarro et al., 2011). Also in microspores, DNA hypomethylation and histone H3 and H4 acetylation have been associated with transcriptional activation and totipotency acquisition (Solis et al., 2012; El-Tantawy et al., 2014; Rodriguez-Sanz et al., 2014b). More recently, the DNA demethylating agent 5-azacytidine, has been reported to induce chromatin decondensation and to promote microspore reprogramming and embryogenesis induction in rapeseed and barley (Solis et al., 2015). Our results indicated that BIX-01294 leads to a reduction in H3K9 methylation which is accompanied by a decrease in global DNA methylation. In animals and plants, an open chromatin structure has been proposed as a requirement to maintain the totipotent state of the cell, “ready for transcriptional activation” (Burton and Torres-Padilla, 2010; Pillot et al., 2010; Gaspar-Maia et al., 2011; Boskovic et al., 2014; Gonzalez-Munoz et al., 2014; Lu and Zhang, 2015; Solis et al., 2015). Taken together, it can be proposed that the reduction in the degree of chromatin condensation (repressive chromatin state) by a transient global decrease in repressive epigenetic marks (H3K9me2 and DNA methylation) would be required for microspore reprogramming and totipotency acquisition. The present work has revealed that during stress-induced microspore embryogenesis, short-term application of the HKMT inhibitor BIX-01294 increased the efficiency of cell reprogramming, totipotency and embryogenesis initiation, together with the reduction of H3K9 methylation. These findings open up new possibilities based on the use of pharmacological strategies that affect HKMT activities to improve the yield of somatic embryogenesis induction, especially in recalcitrant systems.

### Reduction of H3K9 Methylation by BIX-01294 Impairs Microspore-Derived Embryo Differentiation and Development

After microspore reprogramming and embryogenesis initiation, further embryo development involves cell differentiation (Bárány et al., 2010; Solis et al., 2016), a process that is accompanied by silencing of certain gene programs together with expression of cell type-specific programs. Gene silencing during cell differentiation is considered to be associated with formation of chromatin repressive states and heterochromatin, and epigenetic modifications may play a role in the developmental control of gene expression by acting as chromatin modifiers (Grimaud et al., 2006; Köhler and Hennig, 2010; Gutierrez-Marcos and Dickinson, 2012; Eichten et al., 2014). In advanced microspore embryogenesis stages, DNA hypermethylation was related to heterochromatization during cell differentiation (Solis et al., 2012; El-Tantawy et al., 2014). Our results showed progressive increases in H3K9 methylation and *BnHKMT SUVR4-like* gene expression during embryogenesis progression, reaching their maximum levels in cotyledonary embryos. The fact that the presence of BIX-01294 at advanced stages of microspore embryogenesis notably impaired development and reduced embryo production indicates that *de novo* H3K9 methylation is required for differentiation of embryo cells. The assays performed here revealed that BIX-01294 promoted totipotency and embryogenesis initiation in microspores, whereas it clearly prevented further embryo differentiation; the presence of this small compound in the culture medium first increased totipotency and embryogenesis induction efficiency, but later it blocked the process at the proembryo stage. Therefore, the elimination of BIX-01294 at advanced stages, when cell differentiation starts, would favor further development and enhance embryo production. Further work, currently in progress, is necessary to set up a suitable *in vitro* protocol in which BIX-01294 would act at early stages of microspore culture to promote microspore totipotency and enhance embryogenesis induction, whereas BIX-01294 would be removed from the culture at later stages to permit further embryo development.

## Author Contributions

EB performed most of the experimental work of *B. napus* (*in vitro* cultures, immunofluorescence, quantification of H3K9 methylation, qPCR assays, BIX treatments, proembryo and embryo quantification,…) and prepared the figures, IB performed the experiments of *H. vulgare* (*in vitro* cultures, BIX treatments, proembryo quantifications and immunofluorescence), M-TS performed some of the assays of *B. napus* (some qPCR assays, *in vitro* cultures and quantification of H3K9 methylation). YP-P performed the quantification of immunofluorescence signal intensities. MR participated in the design of the work and in the discussion of the results. PT designed and supervised the experimental work, analyzed the results, elaborated the conclusions and wrote the manuscript. All authors read and approved the final manuscript.

## Conflict of Interest Statement

The authors declare that the research was conducted in the absence of any commercial or financial relationships that could be construed as a potential conflict of interest.

## Footnotes

1 http://brassicadb.org
